# Clinical Profile of Triple-Negative Breast Cancer: A Hospital-Based Study

**DOI:** 10.7759/cureus.53373

**Published:** 2024-02-01

**Authors:** Deepak Pankaj, Nitesh Kumar, Anju Singh, Manish Kumar, Zeenat S Imam, Vibhuti Bhushan, Pawan K Jha

**Affiliations:** 1 General Surgery, Indira Gandhi Institute of Medical Sciences, Patna, IND; 2 Pathology, Indira Gandhi Institute of Medical Sciences, Patna, IND; 3 Surgical Oncology, Indira Gandhi Institute of Medical Sciences, Patna, IND

**Keywords:** triple-negative breast cancer, tumor, clinical profile, chemotherapy, breast cancer

## Abstract

Introduction

Triple-negative breast cancer (TNBC) is a new concept and an important area of investigation. In Western country's literature, different studies reported on TNBC and all indicated the poor prognostic aspect of this molecular subtype over other types of breast cancer. However, there is a scarcity of comprehensive data from India. Hence, the present study was carried out to look at the epidemiological and clinical characteristics of TNBC in the Indian population.

Methods

The present study was performed between January 2020 and June 2021 at a tertiary care hospital in Eastern India. A total of 150 patients with TNBC were enrolled in the study. The epidemiological and clinical features of enrolled patients were collected and reviewed.

Results

The median age of patients at TNBC presentation was 45.53 years (24 to 74 years). The median tumor size was reported to be 5.32 cm. Of 150 patients, 94(62.67%) showed enlarged lymph nodes and 56 (37.33%) patients had no lymph node enlargement. In the present study, 85 (56.67%) patients were in the pre/perimenopausal stage at presentation, whereas 65 (43.33%) patients were in the postmenopausal stage. Upon evaluating the spread of TNBC, it was observed that a maximum of patients 60 (40%) were at the T4 stage and 56 (37.33%) at the N0 condition. The clinical staging of TNBC reported a maximum of 74 (49.33%) patients at the IIA, and IIB stages followed by 53 (35.33%) patients at the IIIA, IIIB, and IIIC stages and a minimum of 11 (7.33%) patients at stage IV. Only five (3.33%) patients were reported with a family history of breast cancer. Of all patients, 126 (84%) had detected early breast cancer thereby applicable for surgery at the time of presentation, whereas 71 (47.33%) patients were eligible for radiation therapy and 138 (92%) patients received chemotherapy. A total of 112 (74.67%) patients were found alive after 24 months of follow-up, 22 (4.67%) patients were observed with remission, and 11 (7.33%) patients died due to TNBC progression. During the course of follow-up, five (3.33%) patients were lost in the study.

Conclusion

TNBC is an aggressive malignancy that has a high risk of systemic relapses in the first two years after diagnosis. For more mature evidence on TNBC, longer follow-up of patients is necessary.

## Introduction

Breast cancer (BC) being one of the most widespread cancers among Indian women accounts for around 15% of all cancers [1]. It has already been acknowledged that BC is a varied disease and not a single entity for very long. The conventional histopathologic approaches failed to detect subtypes of breast cancer, which was discovered by gene expression investigations utilizing the DNA microarrays [2]. Four different subtypes of BC have been recognized; these are based on an immunohistochemistry profile, estrogen receptor and progesterone receptor ER/PR, and HER2/neu expression, positive or negative [3]. Also, a fifth subtype called "Normal," has been identified based on gene expression profiling; however, its clinical significance is unknown. Many researchers are of the opinion that it just represents contamination of the tissue sample by normal glandular breast parenchyma [4,5]. 

The definition of basal-like BC has changed throughout time and there is no commonly accepted basis to define it. The panel produced by Nielsen et al. is mostly followed in practice that these basal-like tumors are negative for hormone receptors and human epidermal growth receptor-2 (HER2) and positive for cytokeratin (CK) 5/6 or epidermal growth factor receptor [6]. The basal-type BC more commonly occurs in the younger age group of women who are descendants of African-American origin with poor prognosis and is associated with biological and non-biological factors [7]. The basal-type BC is a more belligerent cancer with relatively small relapse-free survival, an inclination for metastases to viscera rather than bony metastases, and a high chance of BC type-1 mutation susceptibility. Various studies to date on basal-type BC have been restricted by a small sample of study participants and relatively shorter follow-up time. Most of the literature on basal-type BC has been confined to Western literature. To some part, this is because the basal cell-like phenotype is established on antikeratin antibodies used for immunohistochemical staining of tumor slides, which have not been yet used in clinical practice [8]. In clinics, it has been discovered that the "basal-like" category of tumors is almost entirely made up of "triple-negative breast cancer" (TNBC). This TNBC is a tumor that is negative for estrogen receptors (ER), progesterone receptors (PR), and HER2 which can be recognized using immunohistochemistry (IHC) [9]. Hence, IHC has been used as a substitute for identifying basal-like carcinoma in most of the studies around the world. TNBC has attracted pathologists and oncologists' interest as a readily identifiable prognostic category of breast cancer with aggression and a propensity for invasion that often lacks the benefit of any targeted treatment. The prognosis of TNBC from the past to the present has continued to improve due to recent progress in treatment strategies over the existing regime and various recent advances in its management. The overall survival depends on factors such as local, regional, and distant metastasis and the five-year survival rate is not very appealing which ranges from 70 to 80% [10,11]. The reliable data on TNBC in the Indian setting is very less available. Hence, the present study was carried out in Indian settings to find out the clinical profile of TNBC patients in the Indian population. 

## Materials and methods

In the present study, a total of 150 patients with TNBC who were under treatment at Indira Gandhi Institute of Medical Sciences, Patna, Bihar, India between January 2020 and June 2021 were enrolled in the study. The written consent of patients and permission from the Institutional Ethical Committee were taken for the study (1778/IEC/IGIMS/2020). All the participating patients were followed up for 24 months. All diagnosed cases of TNBC who gave their consent were included in the study. Carcinomas other than TNBC of breast were excluded from the study.

Breast cancer was diagnosed mainly by clinical characteristics, various imaging modalities (mammography, ultrasound, or magnetic resonance imaging), and cytopathological tests. Staging for localized disease was done by chest X-ray, abdominal ultrasonography, and computed tomography (CT) scans. The patients were staged as per the American Joint Committee on Cancer (AJCC) staging system. TNBC was considered as ER negative, PR negative, and HER2 negative cancers. These tests were performed using kits approved by the Food and Drug Administration (FDA). Antibody staining of a series of paraffin-embedded slides for estrogen and progesterone receptors was performed for each subject in the database by using a Bio Genex kit. The over-expression of HER-2 was examined by utilizing the Hercep Test kit. HER-2 report of 3+ was taken as positive and a score of 0 or 1 was considered negative in the study. For all 150 patients, baseline epidemiological and tumor features of TNBC were examined. Early breast cancer (EBC) was defined as a T‑stage ≤ T2 and/or N‑stage ≤ N1 and locally advanced breast cancer (LABC) was defined as T‑stage ≥ T3 and/or N-stage≥ N2 without any evidence of distant metastasis. Metastatic breast cancer (MBC) was defined as those breast carcinoma that had confirmed distant metastases. Data analysis and interpretation were performed using Microsoft Excel. The quantitative data obtained were expressed as percentage and numbers in tabular form.

## Results

From the period of January 2020 to June 2021, almost 11.8% of female BC patients were reported to be TNBC on IHC testing of receptor status at our onsite tertiary health care center. Of these, 150 patients fulfilling the inclusion criteria were selected and enrolled for the study. The median age at presentation was 45.53 years, with a minimum age of 24 years and a maximum age of 74 years. 

Of 150 participating patients 85 (56.67%) were pre/peri-menopausal while 65 (43.33%) were reported to be postmenopausal. The median tumor size was reported to be 5.32 cm (3.4 ± 6.2cm).

**Figure 1:**
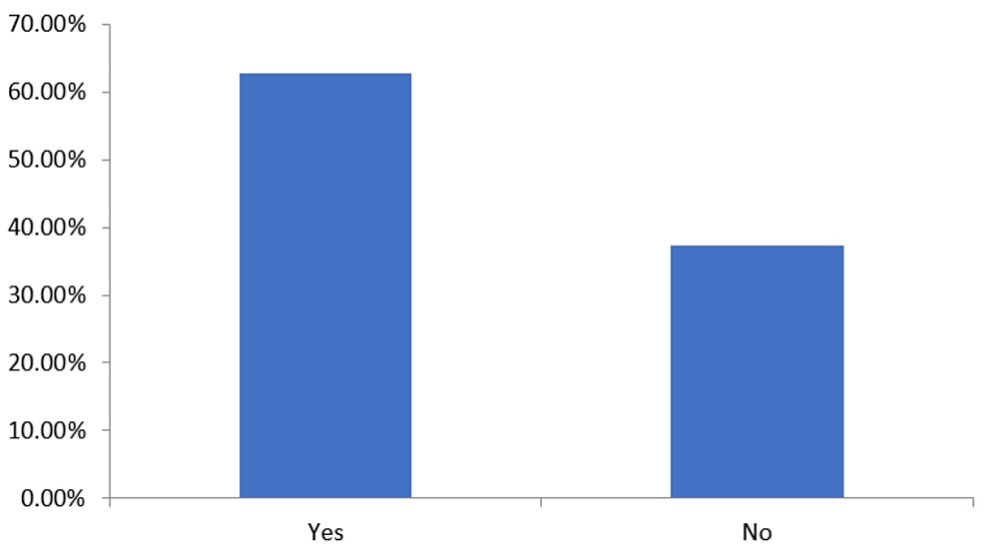
Presence of lymph nodes in patients The data has been represented as percentage (%)

The presence of lymph nodes was examined within the participating patients and it was observed that 94 (62.67%) patients showed lymph node swelling, whereas 56 (37.33%) patients did not show any lymph node swelling (Figure 1).

**Figure 2:**
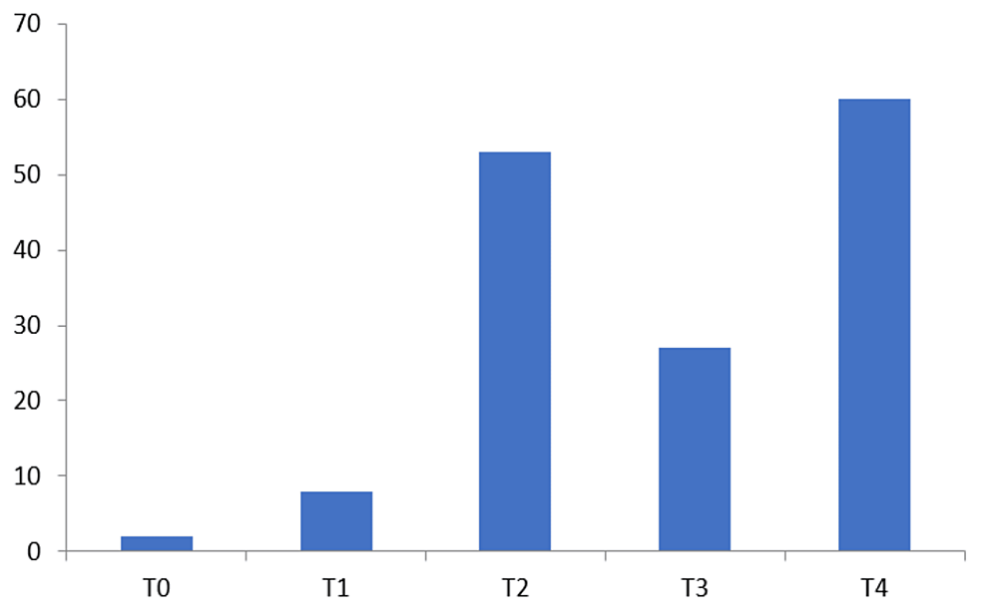
Clinical T stage presentation of participating patients The data has been represented as N (number of patients)

The clinical evaluation of patients revealed that T4 60 (40%) was most common followed by T2 53 (35.33%), T3 27 (18%), and T1 8 (5.33%). The least patients were found at T0 stage 2 (1.33%) in participating patients (Figure 2). There was an insignificant association observed between the size of the tumor and incidences of lymph node positivity.

**Figure 3:**
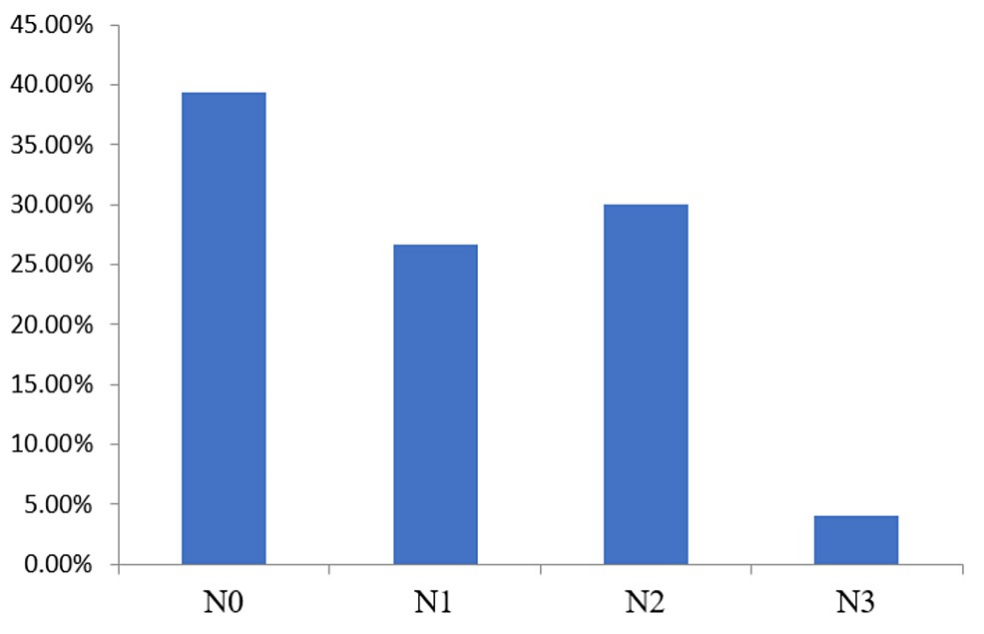
Clinical N stage presentation of participating patients The data has been represented as percentage (%).

Of 150 patients, nodal negative patients were the largest group 56 (37.33%) followed by N2 48 (32%) and N1 40 (26.67%). The least patients were observed at stage N3 6 (4%) (Figure 3). Of 150 participating patients, only five (3.33%) patients had a family history of BC, and remaining patients did not have any BC history. This observation indicated insignificant correlation between family history and observation of TNBC. 

**Figure 4:**
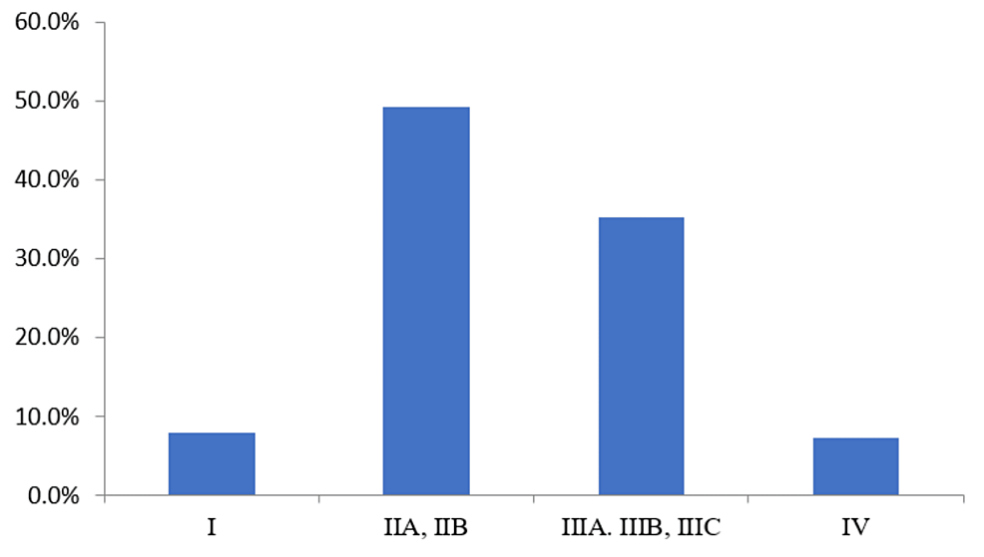
Clinical staging of TNBC of patients The data has been represented as percentage (%). TNBC: Triple-negative breast cancer

Clinical staging of 150 TNBC patients was carried out; it was observed that maximum patients were at stage IIA, IIB 74 (49.3%), followed by stage IIIA, IIIB, and IIIC 53 (35.3%) and stage I 12 (8%), and least patients were reported at stage IV 11 (7.3%) (Figure 4).

**Figure 5:**
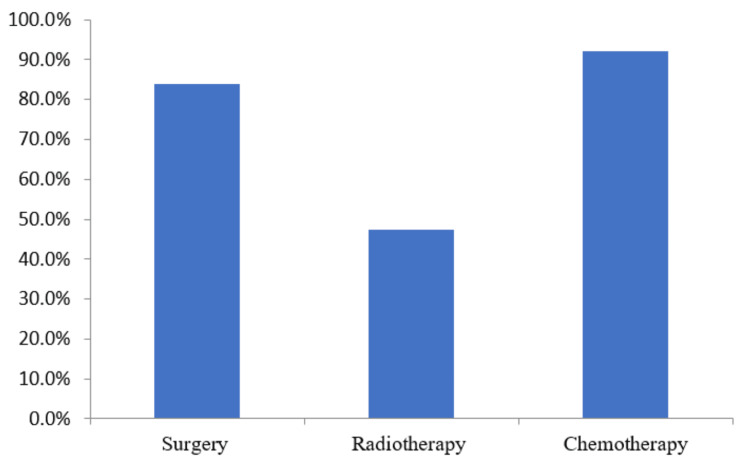
Treatment options of TNBC patients The data has been represented as percentage (%). TNBC: Triple-negative breast cancer

The TNBC among the participating patients was managed by different anticancer treatment techniques. The maximum number of patients 138 (92%) were treated by chemotherapy, followed by surgery 126 (84%), and the least by radiation therapy 71 (47.3%). The results of the TNBC treatment option indicated that most of the patients were at the initial stage of BC (Figure 5).

**Figure 6:**
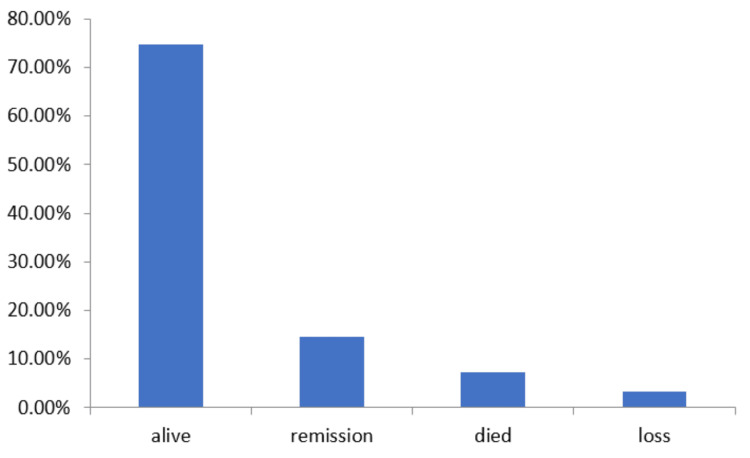
Follow-up observation outcome of TNBC patients The data has been represented as percentage (%). TNBC: Triple-negative breast cancer

The follow-up time was 24 months in the study. There were 11 (7.33%) deaths reported during this follow-up time. All deaths were due to the progressiveness of the TNBC disease. The five (3.33%) patients were lost during follow-up time, all of them were tried to contact by phone but no response was received. The survival rate was found to be 74.67%, a total of 112 patients were reported alive after follow-up. Of 150 patients, 22 (14.66%) patients were observed with remission (Figure 6).

## Discussion

BC is becoming more common across the globe, and in India, it is also a source of worry for treating surgeons and a focus of study. Because of the paucity of Indian data and the higher occurrence of locally advanced BC, the introduction of newer and present-time technology tools that provide insights into tumor pathology is also a significant topic of study. BC holds the highest incidence rate among women in urban India, making it the leading cancer affecting this population., accounting for 15% of all cancers. There is a scarcity of comprehensive and reliable data regarding TNBC in the Indian context. Hence, the present study was carried out in Indian settings to find out the clinical profile of TNBC patients in the Indian population. 

In the current study conducted on the Indian population, the median age of participants was found to be 45.53 years, which is slightly younger compared to the median age of 49 years observed in the Western population. The age range varied from a minimum of 24 years to a maximum of 74 years. This observation of a younger median age in the present study is consistent with the prevailing trend in India, where BC tends to manifest approximately a decade earlier compared to the Western population. This phenomenon highlights the need for early detection and increased awareness among younger women in India to combat this disease effectively.

TNBC was more observed in pre/peri-menopausal females. This finding in the present study agrees with earlier reported studies. However, Khanal et. al. (2020) reported contradictory observations in their study [12].

The TNBC patients in our study exhibited high incidences of lymph node swelling in 94 patients (62.7%). This finding in the present study is in accordance with earlier reported studies.

It is very well documented that TNBC patients usually have large tumor sizes (>2cm) and a high rate of lymph node positivity. Dent et al., who found that even tiny tumors in TNBC had a high likelihood of being lymph node-positive, nicely illustrated this in their research [13]. Our research also discovered that tumor size and lymph node positivity are unrelated, with node positivity in 53 (35.33%) and 27 (18%) patients for T2 and T3 tumors, respectively. Lymph node positivity was seen in eight (5.33%) patients with T1 tumors.

The spread of cancer to nearby lymph nodes in participating TNBC patients was not found significant as a maximum patients 56 (37.33%) patients were reported at the N0 stage and a minimum of patients were reported at the N3 stage 6 (4%). These findings in our study are in accordance with the earlier reported study by Suresh et al. (2013) [14].

The clinical and pathological stage IIA, IIB was observed as the most common stage in 74 patients (49.3%) among participating patients followed by stage IIIA, IIIB, and IIIC in 53 patients (35.3%). The minimum number of patients at stage IV was 11 (7.3%) in the present study which indicated the fewer incidences of invasive BC among participating patients. These findings in the present study agree with other reported studies in the literature.

The present study reported five patients (3.33%) of BC who had a family history. However, Gonzalez-Angulo et al. reported a 19.5% incidence of BC in TNBC patients who had a familial history of BC. There was an insignificant difference observed between family history and observation of TNBC [15].

In our study, treatment of the majority of the patients was managed by chemotherapy 138(92%) and surgery 126(84%) indicating that the incidences of TNBC are at the initial stage in participating patients. Most of the patients in our study were managed by modified radical mastectomy. These observations in the present study are in accordance with the earlier reported study.

In a study conducted by Dent et al., it was demonstrated that patients with TNBC had a higher likelihood of mortality compared to individuals with other forms of breast tumors. The research findings revealed that the mortality rate among TNBC patients was 42.2 percent, whereas it was 28 percent for patients with non-TNBC BC. On average, patients diagnosed with TNBC survived for approximately 4.2 years before succumbing to the disease, whereas those with other types of BC had an average survival time of six years. These findings underscore the critical need for improved treatment strategies and targeted interventions to enhance outcomes for TNBC patients, ultimately reducing the mortality associated with this aggressive form of BC. In the present study, the maximum duration of follow-up was 24 months. There were 11 (7.33%) deaths and 22 (14.66%) relapses among cases reported during the follow-up period. The overall survival rate in the present study was reported to be 112(74.66%), this was in agreement with to study reported by Dent et al. where the survival rate was recorded at 74% in TNBC patients, and by Rakha et al. where the survival rate was observed 83% among TNBC patients [16].

The present study has limitations that it was performed at a single center, which may not be representative of all patients of the Indian population. Furthermore, this study did not give a comparative perspective of TNBC patients with other subtypes of BC patients.

## Conclusions

TNBC is a recognized aggressive tumor, displaying distinct epidemiological, pathological, and clinical outcomes. Our study further highlighted these features, even within a relatively short median follow-up period. We observed a higher proportion of higher-grade tumors among TNBC cases, accompanied by a notable incidence of early relapses. Remarkably, TNBC accounted for approximately 11.8% of breast cancer cases at our tertiary care hospital, primarily affecting younger patients with no remarkable risk factors or family history. Interestingly, no significant association was found between lymph node positivity, tumor size, and the likelihood of high recurrence in TNBC cases. These initial findings emphasize the urgent need for longer follow-up periods to obtain more mature data, which will provide deeper insights into the behavior and management of TNBCs and guide future treatment strategies.
